# Phase separation behavior of whey protein isolate particle dispersions in the presence of xanthan

**DOI:** 10.3906/kim-2004-57

**Published:** 2020-10-26

**Authors:** Alev Emine İNCE COŞKUN

**Affiliations:** 1 Department of Food Engineering, Faculty of Engineering, Ege University, İzmir Turkey

**Keywords:** Colloidal particle, depletion interaction, diffusing wave spectroscopy, flocculation, rod-like depletant, size fractionation

## Abstract

In this study, phase separation of colloidal whey protein isolate (WPI) particle dispersions was studied using a rod-like polysaccharide xanthan. Effects of different xanthan concentration, particle volume fraction, and temperature were analyzed by visual observations, turbidity measurements, and particle mobility tracking method. Particle mobility was determined using a diffusing wave spectroscopy (DWS) set up. Xanthan concentration was kept low in order not to increase the viscosity of dispersions, so that the phase separation could be observed easily. Visual observations showed that there was a minimum concentration of xanthan to induce phase separation at a constant particle volume fraction, and xanthan concentration was found to have an important effect on the degree of phase separation. The temperature was also found to have an effect on depletion mechanism. Phase separation was mainly a result of different sizes of WPI particles, and xanthan induced the depletion interaction between WPI particles, as supported by the data obtained from DWS. The results of this study explained both the mechanism and the stability range of particle dispersions in the presence of xanthan, which is important for the design of stable systems, including colloidal particles.

## 1. Introduction

Proteins are of fundamental importance in food structure and function, and the studies on proteins have always been in the area of biochemistry. In addition, proteins are valuable nutrients for a healthy diet, including muscle growth and weight control [1]. The variety, structure, and characteristics allow proteins a broad use area, particularly in food and pharmaceutical industries.

Whey proteins, constituting a wide range of milk proteins, are highly nutritious and have structuring properties [2]. Above 60 °C, whey proteins undergo a denaturation process [3] and could form a gel network above a certain protein concentration [4]. Therefore, thermal applications to whey proteins could create problems during the processing and storage of foods. The disadvantages of thermal applications, such as an increase in the turbidity or viscosity of liquids, could be eliminated using the particulate form of whey proteins, and thereby allowing a better control over the stability of food products. Recently, formulations of protein particles from different sources with different characteristics have attracted an increasing interest [5-7]. Such colloidal particles can be used in structuring of foods or in controlled delivery systems. To form protein particles, several gelation techniques, such as heat-induced [8], salt-induced [9], pH-induced [10], and enzymatic cross-linking [11] can be used. Among these techniques, heat-induced gelation is the most commonly used technique. 

Polysaccharides are widely used in foods as thickener, stabilizer, or emulsifier. The rheological behavior of solutions can be modified by using different polysaccharides [12]. Xanthan is a negatively charged, non-gelling polysaccharide with a linear backbone of 1,4-linked b-D-glucose with two side chains [1] and has a radius of gyration of 241 nm [13]. Xanthan could create viscous solutions, however, to induce gelation, it is often combined with other polysaccharides, such as carrageenan or locust bean gum [14,15]. Due to its thickening property, xanthan is widely used in salad dressing, sauces, and bakery products [14,16]. 

Interaction of whey proteins and xanthan could be electrostatic. Xanthan is negatively charged at all pH values [1], whereas whey proteins have an isoelectric point of around pH 5.2 [17]. Therefore, above pH 5.2, no electrostatic attraction has been expected as both whey proteins and xanthan are negatively charged, whereas below this point, they attract each other and could form complex coacervates [18]. 

Another type of interaction between whey protein particles and xanthan is the depletion interaction. Depletion interaction between non-interacting colloidal spheres and rod-like polymers in aqueous mixtures, which was first discussed in 1958 [19], has already been studied widely [20,21]. Depletion interaction between two large spheres (R) was reported to be caused by rod-like depletants with much smaller values in length (L) and diameter (D), D<<L<<R [20]. Polymers, due to their smaller size than the colloidal particles, create void volumes, and therefore the osmotic pressure between particles induces flocculation. Once the osmotic pressure is sufficiently high, the depletion layers between particles overlap, and they start to form aggregates [22,23]. The size and volume fraction of particles, and the size and concentration of the depletant are important factors affecting the depletion-induced aggregation [24]. 

Whey proteins and xanthan are widely used in liquid food products. The presence of these two biopolymers in the same medium may constitute a phase separation problem during the processing and storage of products. Therefore, the objective of this study is to gain insight into the mechanism behind the stability of dispersions. A detailed stability analysis of colloidal WPI particles in the presence of xanthan is new in the literature. The fractionation of WPI particles with respect to their size in the presence of a depletant would create monodisperse systems, which are easier to control and yield more stable emulsions or dispersions. In this study, the phase separation behavior of WPI particle dispersions in the presence of xanthan was investigated mainly according to the parameters in Stoke’s law. According to Stoke’s law, the velocity (V) of a spherical particle in dispersion is given by

V=2(ρp-ρf)gR2/(9μ)

where ρ_p_ is the density of particles, ρ_f_ is the density of aqueous phase, *g* is the gravity, R is the radius of the particle, and µ is the viscosity of aqueous phase [25]. Influences of xanthan concentration and particle volume fraction have been expected to increase the viscosity of the aqueous phase and were analyzed by determining the phase separation through a turbidimetric analysis. Viscosity of the aqueous phase was determined, and the mobility of the particles, which could be suppressed by the increasing viscosity, was measured using diffusing wave spectroscopy (DWS). The difference in phase separation behavior of dispersions at 4 °C and 60 °C was also discussed to determine whether there was a thermodynamic contribution. 

## 2. Materials and methods

### 2.1. Materials

Whey protein isolate (BiPro, Lot 198-1-420, Davisco Foods International Inc., Minnesota, USA) was used to prepare protein particles. The composition of whey protein isolate (WPI) was given as 90.25% protein, 0.95% fat, 2.85% ash, 0.95% lactose, and 5% moisture. Polyglycerol polyricinoleate (PGPR) was obtained from Danisco DuPont (GRINDSTED® PGPR, Denmark). Sunflower oil (Reddy, NV Vandemoortele, Breda) was purchased from a local supermarket. Xanthan gum from *Xanthomonas campestris* was obtained from Sigma-Aldrich (Steinheim, Germany) in yellowish powder form. According to the supplier’s report, the viscosity value of 1% xanthan solution was in between 800 cps and 1200 cps.

### 2.2. Preparation of WPI particles, their dispersions, and visual observations

WPI particles were prepared by using a two-step emulsification method [6]. In this method, a 25% (w/w) WPI solution (aqueous phase) was emulsified in a 2.5% (w/w) PGPR solution (oil phase). A 30% (w/w) water-in-oil emulsion was prepared by the addition of aqueous phase to the oil phase slowly using a high-speed blender (Ultraturrax, T25 Digital, IKA Werke, Germany). The emulsion was heated at 80 °C for 20 min for the gelation of droplets to produce protein particles. Then, the emulsion was cooled to room temperature and centrifuged (34000 RCF, Avanti J-26 XP, Beckman Coulter, USA) for 1 h to remove the oil. The obtained pellet was washed with a 1% (w/w) WPI solution with a 1 / 2 (w/w) pellet to WPI solution ratio, to remove the remaining oil between the particles. The washing was done by redispersion of the pellet using a high speed blender (12800 rpm for 5 min) and a homogenizer (LabhoScope Homogeniser, Delta Instruments, Drachten, The Netherlands) at 150 bar for 6 cycles. Then, the dispersion was centrifuged again, and the washing step was repeated twice. Final dispersions of the WPI particles were prepared in 1% (w/w) WPI solution at the desired volume fractions. 

A 2 g/L stock xanthan solution was prepared in distilled water, and then it was added to the dispersions. Visual observations were done daily for 4 days either at 4 °C (the samples were kept in a refrigerator) or at 60 °C water bath. The pH of the dispersions was around pH 7, and no pH adjustment was done. 

Visual observations were reported either as pictures or on graphs that showed the relative sedimentation height, which was defined as the sedimentation height divided by the total sample height in the tube. Relative sedimentation height could be used as a way of showing the degree of phase separation, which indicates the strength of forces acting on the particles.

### 2.3. Determination of volume fraction

The hydrodynamic volume fraction (Φ) of the particles was determined using Einstein’s viscosity equation:

ηeff=ηc(1+2.5Φ)

where η_eff_ is the dynamic viscosity of the dispersion, and ηc is the dynamic viscosity of 1% (w/w) WPI solution as continuous phase. A capillary viscometer (Ubbelohde, 0B 39709) was used at 20 ± 0.05 °C to measure the dynamic viscosity of sufficiently diluted dispersions. All the measurements were repeated at least twice.

### 2.4. Determination of particle size distribution

Particle size distribution is a direct method to determine the differences in particle sizes in the upper and lower parts of dispersions upon phase separation and to determine the polydispersity of particles via the shape of the size distribution curve. A multi angle light scattering instrument (Mastersizer 2000 Malvern Instruments, Worcestershire, U.K.) equipped with a distribution unit (HydroSM 2000A, 1200 rpm) was used. The dispersions were diluted with distilled water. Each measurement was repeated at least twice, and average values were reported.

### 2.5. Determination of viscosity

Zero shear viscosity of dispersions were determined using a rheometer (Physica MCR 501, Anton Paar, Graz, Austria) equipped with a concentric cylinder geometry (C-CC17/TI-SN39511). The shear rate was logarithmically increased from 1 s^-1^ to 1000 s^-1^ at 20 °C. Each measurement was repeated twice, and average values were reported.

### 2.6. Determination of turbidity profiles

Stability of dispersions against sedimentation was determined using LUMiFuge (1120-34 (8 channels) LUM GmbH, Berlin, Germany), which accelerates the destabilization of samples by applying a centrifugal force. LUMiFuge basically measures the transmission of light, and therefore the turbidity of samples can be determined sensitively. During the measurement of transmission through the length of cell, LUMiFuge applies a centrifugal force to fasten the destabilization of the dispersions [26]. According to its working principle, the opaque part of the samples yields low transmission profiles, whereas the transparent part of the samples yields high transmission profiles. The wavelength was 870 nm, and the temperature was 20 °C. Sample cells (LUM 2 mm, PC, Rectangular Synthetic Cell (110-131xx), Berlin, Germany) with 2.2 mm optical path were used. The samples were subjected to a centrifugal force of 20 g (413 rpm) and measured every 10 min for 200 min. Normalized transmissions were calculated at a certain position of the cell by dividing the transmissions at different times to the initial transmission of the sample. All the measurements were repeated twice.

At a certain volume fraction (Φ ~ 0.05) of particles, the turbidity analysis was performed with increasing xanthan concentrations up to 1 g/L in the aqueous phase. This value was determined with preliminary experiments, at which the viscosity of the system did not highly increase, and therefore one can interpret the effect of the depletion interaction easily. Similarly, the effect of the volume fraction of the particles in terms of turbidity profiles was analyzed at a certain xanthan concentration (0.05 g/L), which was chosen from the concentration values below 1 g/L and where the phase separation was known to occur.

### 2.7. Diffusing wave spectroscopy (DWS)

WPI particle dispersions were analyzed at room temperature using a DWS set up in transmission mode. DWS is able to track the mobility of particles and give information about the aggregate formation and the diffusivity of systems [27]. Autocorrelation function g2(τ), which is related to the motion of the particles, was measured [28]. Intensity autocorrelation function is given by

g2(τ)=(∫0∞P(s)e-((s/l*)k2‹Δr2(τ)›)ds)2

where P(s) is the path lengths distribution function of the photons, *l* ^*^ is the transport mean free path, k is the wave vector of the light, and 〈Δ *r* ^2^(τ)〉 is the mean square displacement. The half-life time (τ_1/2_) was defined as g_2_(τ_1/2_) – 1 = 0.5 [29]. A He-Ne laser (20 mW) was used at a wavelength of 633 nm. The beam was expanded to 2 mm. A transparent glass cell ( *l* = 2 mm) was used. The beam was split into two photomultiplier tubes (ALV/SO-SIPD, Germany), which converted the optical intensity into pulses. Then, a digital correlator (ALV-5000/60X0, Germany) created the autocorrelation function. Each measurement was done for 30 s for every 10 min in 1 h.

The data set for DWS was the same as those for turbidity measurements; which were increasing xanthan concentration up to 1 g/L at a constant particle volume fraction of 0.05 and increasing particle volume fractions from 0.01 to 0.1 at a constant xanthan concentration, 0.05 g/L. Data obtained from DWS can be interpreted as a set of supporting data for the effect of viscosity on sedimentation behavior.

### 2.8. Statistical analysis

The experiments for the statistical analysis were done in duplicates. MINITAB 17.0 (Minitab Inc. State College, PA, USA) was used for all the statistical analyses. The pairwise comparisons were made by Tukey’s test with a significance level of 0.05. 

## 3. Results and discussion

### 3.1. Effect of xanthan concentration

**Figure 1:**
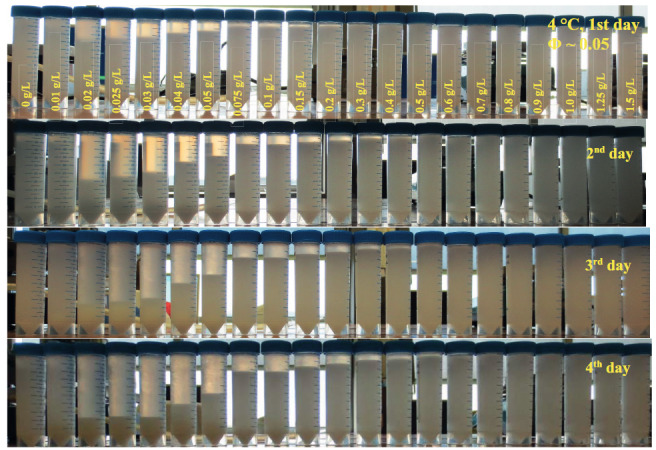
Pictures of WPI particle dispersions (Φ ~ 0.05) with different xanthan concentrations from 0 g/L to 1.5 g/L at 4 °C for 4 days.

Figure 1 shows the pictures of WPI particle dispersions (Φ ~ 0.05) at different xanthan concentrations from 0 g/L to 1.5 g/L at 4 **°** C on different days. This picture shows that a sediment layer started to be formed in dispersion with 0.02 g/L xanthan at 4 **°** C at the 1st day of the preparation. There is a gradual increase in sediment height up to 0.4 g/L xanthan. Above 0.5 g/L xanthan, dispersions showed no phase separation; therefore, one can consider them as stable. A similar phase separation behavior was previously reported for sodium caseinate solutions in the presence of xanthan, and they attributed the reason to segregative microphase separation which stemmed from thermodynamic incompatibility at neutral pH as a result of repulsive electrostatic interactions between xanthan and the protein [30,31]. In addition, previous research reported that there was a critical concentration of depletant required to induce the aggregation of particles, which is in line with the findings of the observation of WPI particle dispersions [32]. 

**Figure 2:**
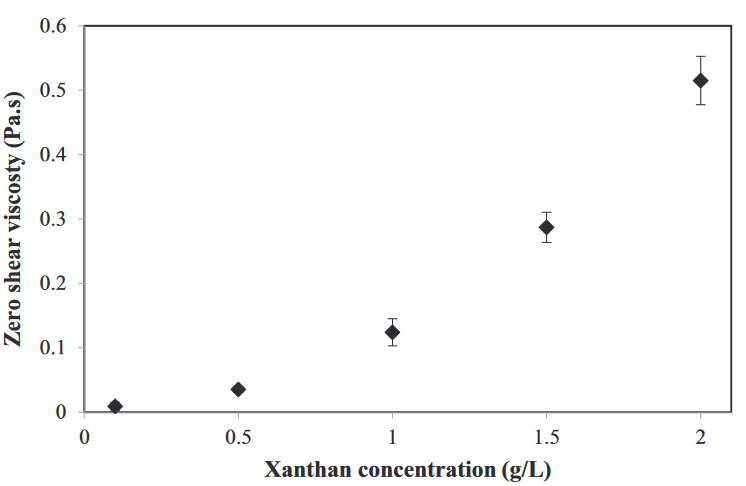
Zero shear viscosity of different xanthan concentrations in 1% (w/w) WPI solution (n = 2).

It is known that xanthan is a non-gelling hydrocolloid [14]; however, it is able to highly increase the viscosity of a solution and gives a pseudoplastic characteristic to the solution [30]. Above 0.5 g/L xanthan, as all the dispersions seem to be stable (Figure 1), one may think that the viscosity increase contributed to the stability. Therefore, the zero shear viscosity of 1% (w/w) WPI solutions at different xanthan concentrations, which was actually the aqueous phase of the dispersions, was determined. In Figure 2, the viscosity of WPI solutions with increasing xanthan concentrations is presented. Increasing xanthan concentrations were found to increase the viscosity of solutions, which is in line with previous research [30]. Particularly, above 1 g/L xanthan, the viscosity increase was more pronounced. It is known that the mobility of the colloidal particles could be limited by increasing the viscosity of the aqueous phase according to Stoke’s law [33]. Therefore, to understand the mechanism behind the destabilization, phase separation of the systems was induced by keeping the xanthan concentration low. 

**Figure 3:**
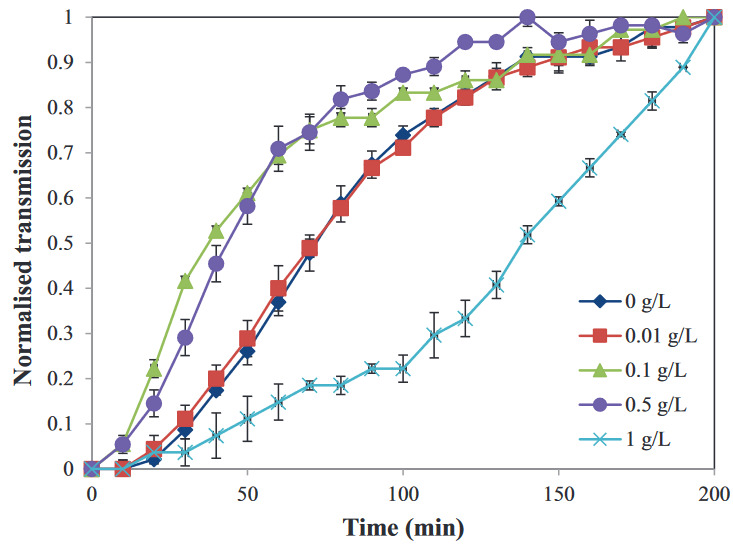
Normalized transmission curves of WPI particle dispersions (Φ ~ 0.05) with different xanthan concentrations ((♦) 0 g/L, (■) 0.01 g/L, (▲) 0.1 g/L, (●) 0.5 g/L, and (×) 1 g/L xanthan) under 20 RCF (n = 2).

The destabilization of WPI particle dispersions at a volume fraction, Φ 0.05 with increasing xanthan concentrations from 0 g/L to 1 g/L were analyzed by using LumiFuge. Figure 3 shows the normalized transmission profiles of WPI particle dispersions (Φ ~ 0.05) with different xanthan concentrations for 200 min. According to this graph, dispersions with no and 0.01 g/L xanthan show almost the same transmission profile, and the dispersions with 0.1 g/L and 0.5 g/L xanthan show higher transmission profiles compared to the previous ones. This indicates that the phase separation of dispersions with 0.1 g/L and 0.5 g/L xanthan occurred faster under 20 RCF. The dispersion with 1 g/L xanthan showed lower transmission profiles, which indicated higher dispersion stability than the others, possibly due to the viscosity increase in the aqueous phase. All the dispersions, except the one with 1 g/L xanthan, started to level off after ~ 2 h, which showed that the destabilization of these dispersions was mainly induced less than 2 h under 20 RCF. The trend of phase separation behavior at the concentrations used was in line with the visual observations. 

Phase separation of dispersions often occurs as a result of strong electrostatic attraction leading to bridging flocculation [32]. However, the pH value of all the samples was at pH 7, and therefore no attractive interaction was expected between WPI particles and xanthan as they were both negatively charged [16,30]. In this case, the type of interaction between WPI particles and xanthan was likely to be steric rather than electrostatic [34,35]. 

Xanthan could induce depletion flocculation at low concentrations, whereas at high concentrations, it could increase the stability of dispersions by increasing the viscosity. Xanthan and WPI particles hold the criteria of being much smaller rod-like depletant and larger colloidal spheres (D<<L<<R) to induce the depletion interaction [20], as the gyration radius of xanthan was ~ 0.25 µm [13], and the size of the WPI particles were measured as ~ 3 µm. Previously, the effect of xanthan on the physical stability of oil-in-water emulsions was investigated, and above a certain concentration of xanthan, depletion flocculation was found to occur [36]. Furthermore, in the same study, increasing xanthan concentrations slowed down the flocculation rate of emulsions, and the reason was attributed to two possibilities: one was the increasing viscosity of the emulsion due to increasing xanthan concentration, and the other was the occurrence of phase separation at the microscopic level [37]. In the second case, as a result of depletion flocculation, a gel-like network structure was expected to form [36]. Similar to the oil droplets in an aqueous medium, soft WPI particles underwent a depletion-induced aggregation in the presence of xanthan.

**Figure 4:**
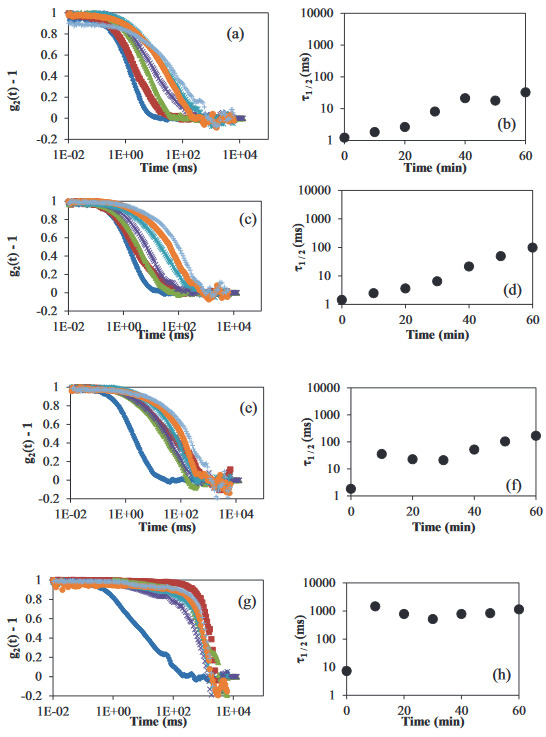
First column shows the normalized g2(t) – 1 curves of WPI particle dispersions (Φ ~ 0.05) with (a) 0 g/L, (c) 0.01 g/L, (e) 0.1 g/L, (g) 0.5 g/L xanthan at different times; (♦) 0 min, (■) 10 min, (▲) 20 min, (×)30 min, (*) 40 min, (●) 50 min, (+) 60 min. The second column shows the half-life times of WPI particle dispersions (Φ ~ 0.05) with (b) 0 g/L, (d) 0.01 g/L, (f) 0.1 g/L, (h) 0.5 g/L xanthan.

To gain insight into the stability mechanism of the WPI particle dispersions (Φ ~ 0.05) with different xanthan concentrations, diffusing wave spectroscopy (DWS) was used. Decorrelation curves are generated by tracking the displacement of particles; therefore, DWS could give information about the diffusivity or the particle kinetics in a dispersion system [27]. Figure 4 shows the decorrelation curves and corresponding half-life times of WPI particle dispersions (Φ ~ 0.05) with increasing xanthan concentrations from 0 g/L to 0.5 g/L. Figure 4a and 4b correspond to the WPI particle dispersion without xanthan. A gradual increase in decorrelation time can be seen (Figure 4a). Half-life times of the dispersion shifted to higher values in 1 h (Figure 4b), indicating a kinetic change in the particle movement. The shift to higher decorrelation times for this sample is possibly due to the sedimentation of large colloidal particles. Although sedimentation was not observed visually during 4 days (Figure 1), the particle kinetics implied that the sedimentation had started. The dispersion with 0.01 g/L xanthan followed the same trend in the case of no xanthan (Figure 4c). Half-life times (Figure 4d) shifted to larger values compared to the sample without xanthan. This increase in half-life times was presumably an indication of the decrease in the mobility of the protein particles [27]. For these two samples, the similar sedimentation behavior was obtained from LumiFuge. Previously, at very low depletant concentrations, the entropy change correlated to the particle aggregation was reported to overcome the depletion interaction, leading to a stable system [38]. This means that the depletion interaction was still there, but was not sufficiently high to induce a phase separation. In Figure 4e, where there was 0.1 g/L xanthan in the dispersion, decorrelation curves shifted to large values and overlapped after the first curve. Correspondingly, the half-life times level off after the first data point (Figure 4f). The overlapped curves indicate the stability of dispersion over the time of measurement. The dispersion became unstable directly after the mixing process, possibly due to the flocculation of the particles as a result of the depletion-induced attraction. Visual observations of this sample showed a clear phase separation in 24 h, which was faster than the one in the cases of other xanthan concentrations. This indicates that the strength of the depletion forces was higher in the dispersion with 0.1 g/L xanthan. Figure 4g shows the decorrelation curves, and Figure 4h shows the half-times of the dispersion with 0.5 g/L xanthan. The shape of the decorrelation curves is different from the samples with low xanthan concentrations, which indicates the kinetics of the particles changed. Half-life times are higher, and the increase toward these values is much steeper than that in the cases of lower xanthan concentrations. The increase of half-life times could indicate a decrease in the mobility of particles [27]. The dispersion was also visually stable, and almost no phase separation was observed (Figure 1). A similar observation for the stability of latex dispersions in the presence of WPI fibrils was also made by others [29]. They reported that the stabilization mechanism depended on the fibril concentration, which altered the kinetic barrier. Therefore, the reason for the stability can be attributed to the depletion stabilization. The other possibility is the viscosity increase in the aqueous phase; however, as the zero shear viscosity values show only a smaller difference (Figure 2) between 0.1 g/L and 0.5 g/L xanthan, depletion stabilization is likely to happen.

Increasing xanthan concentrations at the same particle volume fraction changed the kinetics of particles, thereby changing the stability of the bulk system. The dispersion without xanthan showed the sedimentation under gravity, which was an expected situation according to Stoke’s law. A xanthan concentration of 0.01 g/L in dispersion was probably below the critical concentration to induce phase separation [32], as the dispersion was visually stable; however, the mobility of the particles indicated a change compared to the dispersions without xanthan. The phase separation of the dispersion with 0.1 g/L xanthan was a result of depletion flocculation, and the stability of the dispersion with 0.5 g/L xanthan was probably due to the depletion stabilization, as the effect of rods in colloidal suspensions was previously reported to increase the energy barrier for flocculation [20]. At all xanthan concentrations, whether the dispersions were stable or unstable, depletion interaction seems to play an important role in their phase separation behavior. 

### 3.2. Effect of particle volume fraction

**Figure 5:**
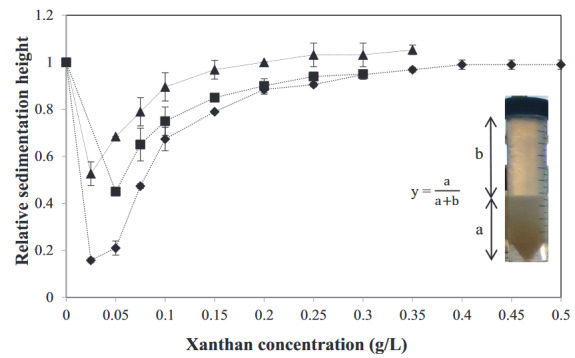
Relative sedimentation height of WPI particle dispersions at (♦) 0.05, (■) 0.1, and (▲) 0.15 volume fractions at different xanthan concentrations after 4 days of preparation (n = 2).

Visual observations of dispersions at three different volume fractions with increasing xanthan concentrations were made, and the relative sedimentation height is shown in Figure 5. These data were obtained on the 4th day of the observation, when the dispersions reached the equilibrium. According to the graph, at the same xanthan concentration up to 0.25 g/L, the phase separation at low volume fractions (Φ ~ 0.05 and Φ ~ 0.1) was more pronounced. This means the dilute dispersions can be destabilized more quickly with the same amount of xanthan. From a different perspective, above 0.5 relative sedimentation height, less xanthan was required to induce destabilization for the same degree of sedimentation for concentrated particle dispersions. Similarly, in a previous study, a decrease in critical polymer concentration to induce sedimentation of dispersions with increasing particle volume fractions was reported [38]. Dispersions with 0.05 g/L and 0.075 g/L xanthan at different volume fractions showed a fast sedimentation on the first day of the preparation. This indicates that the depletion forces were sufficiently high to induce the phase separation. At the xanthan concentrations between 0.15 g/L and 0.25 g/L, an increase in the sedimentation height was observed, which means the depletion forces to induce phase separation decreased. At xanthan concentrations above 0.3 g/L, depletion forces could have turned into a stabilizing effect. In addition, at these xanthan concentrations, viscosity increase could also have an effect on the dispersion stability.

**Figure 6:**
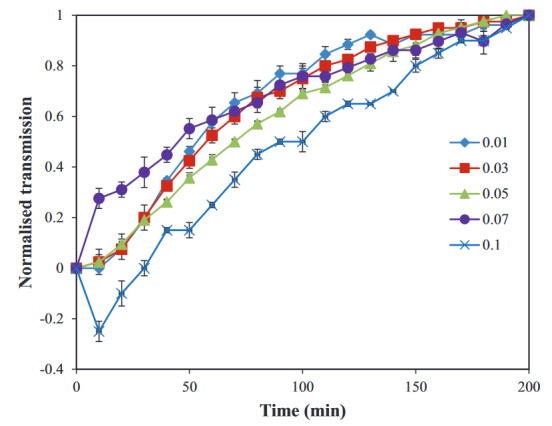
Normalized transmission curves of WPI particle dispersions with 0.05 g/L xanthan at different volume fractions, (♦) Φ ~ 0.01, (■) Φ ~ 0.03, (▲) Φ ~ 0.05, (●) Φ ~ 0.07, and (×) Φ ~ 0.1 under 20 RCF (n = 2).

To see the effect of volume fractions more sensitively, turbidity analysis was done using LumiFuge. In Figure 6, normalized transmission profiles of dispersions with 0.05 g/L xanthan at different volume fractions are shown. Transmission profiles are close to each other, and some of them seem to overlap. At the same xanthan concentration, dispersions seemed to show similar behavior at different volume fractions. This finding suggests that the volume fraction is less effective on the phase separation behavior of the dispersions compared to the effect of xanthan concentration. As previously stated in the theory of depletion flocculation, the concentration of the depletant is of great importance [32].

**Figure 7:**
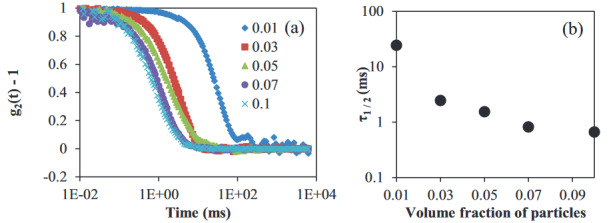
(a) Normalized g2(t) – 1 curves of WPI particle dispersions with 0.05 g/L xanthan at different volume fractions, (♦) Φ ~ 0.01, (■) Φ ~ 0.03, (▲) Φ ~ 0.05, (●) Φ ~ 0.07, (×) Φ ~ 0.1, and (b) half-life times of WPI particle dispersions with 0.05 g/L xanthan at different volume fractions.

Figure 7 shows the decorrelation curves (Figure 7a) and half-life times (Figure 7b) of dispersions at different volume fractions. Decorrelation curves shifted to lower values with increasing volume fraction, indicating the mobility of the particles changed. At higher volume fractions, the visual observations showed an increased stability of the dispersions (Figure 5). Thus, the decorrelation curves should indicate a different mobility of particles at higher volume fractions, which resulted in more stable dispersions. As the stability of dispersions was favored at higher volume fractions, the particle volume fraction seems to have an effect on the stability of dispersions. Previously, the dilute oil-in-water emulsion was found to cream faster in the presence of a surfactant micelle (SDS), and the creaming rate was reported to decrease with the increasing volume fraction of droplets [39]. The reason for the creaming rate was attributed to the depletion flocculation when the depletant concentration was above a certain limit. In another study, in oil-in-water emulsion with a non-gelling hydrocolloid in the aqueous phase, higher destabilization of emulsions by flocculation was reported when the oil volume fraction was low [38]. Similar findings were obtained in the case of WPI particle dispersions. Therefore, the effect of depletion flocculation for the destabilization mechanism of the WPI particle dispersions at low volume fractions cannot be ignored.

More dilute dispersions, for which the particle volume fractions were smaller, showed a faster phase separation at the same xanthan concentration. Sensitive light scattering techniques also supported this finding although no phase separation has been observed visually yet. Presumably, the micro-phase separation conditions were hold [30]; however, there was not enough time for the visible phase separation. 

### 3.3. Effect of different temperatures

Depletion interaction is often known as changing the kinetic stability of particles in dispersions [32]. To see the thermodynamic effect on the phase separation, dispersions at 4 °C and 60 °C were analyzed by visual observations and determining the particle size distributions. The behavior of samples at 4 °C was already discussed in previous sections (Figure 1). According to Figure 1, the dispersion without xanthan showed only sedimentation due to gravity. At 0.01 g/L xanthan, the depletion interaction was possibly too low to induce flocculation and a clear phase separation. The depletion interaction was more pronounced starting from 0.02 g/L up to 0.4 g/L. The strength of the depletion forces was high in the dispersion with 0.02 g/L xanthan, as it showed a quick phase separation on the first day of the preparation. Gradual increase in the sediment height up to 0.4 g/L xanthan indicates the decreasing in the strength of depletion interaction between particles. Above 0.5 g/L xanthan, dispersions were stable, as already stated.

**Figure 8:**
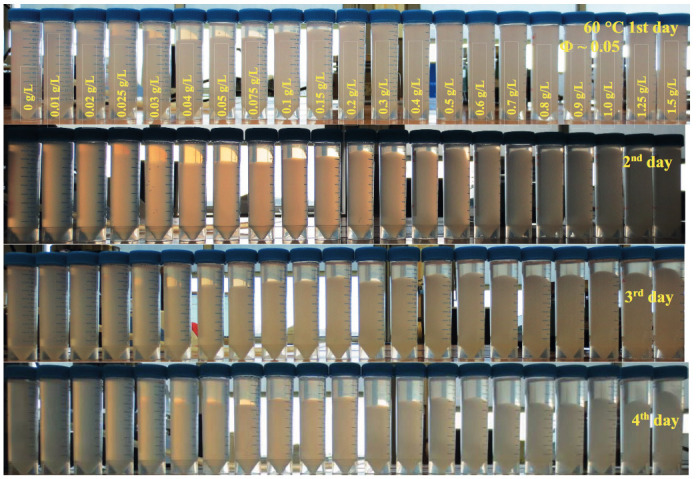
Pictures of WPI particle dispersions (Φ ~ 0.05) with different xanthan concentrations from 0 g/L to 1.5 g/L at 60 °C for 4 days.

Figure 8 shows the WPI particle dispersions with different xanthan concentrations at 60 °C. The appearance of the dispersions at 60 °C was translucent, which could be an indication of the swelling of the particles [40]. On the 1st day of preparation, phase separation observations were similar to the ones in 4 °C; in other words, the dispersions starting from 0.02 g/L up to 0.4 g/L xanthan concentration showed phase separation. On the 4th day of the preparation, the phase separation behavior shifted to the higher xanthan concentrations compared to the samples at 4 °C. The clear upper phase was observed for all the dispersions with xanthan concentrations above 0.04 g/L. This suggests that a thermodynamic effect applies to the phase separation behavior of dispersions. Temperature increase changed the phase separation behavior of the dispersions at 60 °C compared to the dispersions at 4 °C. The viscosity of xanthan solutions at high temperatures is known to decrease [41], which enables the destabilization of the system. In addition, the swelling of the particles [40] could have also fastened the sedimentation. These results possibly led to phase separation of the dispersions even at higher xanthan concentrations.

The size distribution of the particles is shown in the Table. The volume averaged particle sizes at 4 °C was around 3 µm, and the size of particles at 60 °C was measured around 7 µm (Table). Unless otherwise indicated, the size distribution of particles was measured from the lower phase of the dispersions. These data indicate the swelling of particles at increasing temperatures and possibly the presence of additional effects, such as aggregation or falling apart of the particles. The dispersions were prepared at a volume fraction of 0.05 at room temperature with an average diameter of 2.8 µm without the addition of xanthan. In this case, each particle can maximally be 6.4 µm, as the closed-pack system (Φ ~ 0.6) would be reached [42]. Therefore, the averaged diameters, which were above 6.4 µm, indicate that other factors contribute to the increase in size, such as aggregate formation or accumulation of proteins around the particles. Alternatively, some particles may have not survived at 60 °C and may have fallen apart. In this case, the average diameters of particles could increase up to measured values without reaching the closed-pack conditions. In anyway, the particle size increase at 60 °C showed that there were additional effects, being different from swelling.

**Table:** Volume-averaged mean diameters of WPI particles in the presence of different concentration of xanthan (n = 2).

Xanthan concentration in WPI dispersion (g/L)	Volume averaged mean diameter,D [4,3], of particles (µm)	
	At 4 °C	At 60 °C
0	2.75 ± 0.07^ABb^	7.65 ± 0.07^ABa^
0.02 (upper phase)	1.10 ± 0.00^C^	-
0.02 (lower phase)	2.95 ± 0.07^Ab^	7.75 ± 0.07^Aa^
0.05	2.90 ± 0.00^ABb^	7.30 ± 0.00^BCa^
0.1	2.75 ± 0.07^ABb^	7.15 ± 0.07^Ca^
0.5	2.70 ± 0.00^Bb^	7.10 ± 0.00^Ca^
1.0	2.85 ± 0.07^ABb^	6.15 ± 0.21^Da^

Data in the same column with different uppercase superscript letter and data in the same row with different lowercase superscript letter are significantly different (P ≤ 0.05).

Protein particles were obtained with a certain polydispersity; however, the polydispersity range can be narrowed down by a size fractionation method. The measurement of the particle sizes in the upper and lower phase of dispersion with 0.02 g/L xanthan at 4 °C shows that the size fractionation of polydisperse colloidal particles using xanthan was possible (Table). For other xanthan concentrations, size measurement could not be performed to the upper phase of dispersions. The size fractionation of WPI particles in the dispersion at Φ ~ 0.05 and at 0.02 g/L xanthan could be specific to the size ratio of the two biopolymers. 

Statistical analysis showed that there is not a significant difference between the sizes of the particles at 4 °C, whereas there are considerable differences between those at 60 °C. This result demonstrates that the particles at 4 °C did not form nonsoluble aggregates, which indicates that the strength of the depletion interaction was not high. On the other hand, particle size at 60 °C demonstrates a decreasing trend with increasing xanthan concentration. This could be a result of macromolecular crowding, which probably suppressed the swelling of the particles [35,43]. 

The presence of xanthan, as a depletant, induced phase separation of dispersions at both 4 °C and 60 °C; however, the concentration of xanthan to induce destabilization differed. At high temperatures, the mobility of particles increases, and viscosity of aqueous phase decreases. These effects made the destabilization of the systems easy to occur. In addition to these facts, the presence of xanthan induced a faster phase separation, as a result of the depletion interaction between particles. 

## 4. Conclusion

In this study, the presence of xanthan was found to induce phase separation of whey protein isolate (WPI) microparticle dispersions. The mechanism behind the phase separation was determined as the depletion interaction between the particles. The effect of depletion interaction was analyzed using turbidity measurements and particle tracking methods, both of which showed that the depletant concentration had an important effect on the phase separation behavior. The use of these two methods for the stability analysis of colloidal dispersions constitutes the novelty of this research. The results of this study revealed the mechanism of phase separation of WPI dispersions in the presence of xanthan. Furthermore, it was shown that a food-grade biopolymer, xanthan, could be used to fractionate polydisperse systems to create more monodisperse systems. In this way, emulsion and dispersion systems with high stability could be obtained. Further studies may include the electrostatic interactions between depletants and colloidal particles, which could enhance the stability of dispersions against aggregation or phase separation. 
